# Emerging Roles of Vitamin D-Induced Antimicrobial Peptides in Antiviral Innate Immunity

**DOI:** 10.3390/nu14020284

**Published:** 2022-01-11

**Authors:** John H. White

**Affiliations:** Department of Physiology, McGill University, Montreal, QC H3G 1Y6, Canada; john.white@mcgill.ca

**Keywords:** Vitamin D, antimicrobial peptides, antiviral immunity, COVID-19

## Abstract

Vitamin D deficiency, characterized by low circulating levels of calcifediol (25-hydroxyvitamin D, 25D) has been linked to increased risk of infections of bacterial and viral origin. Innate immune cells produce hormonal calcitriol (1,25-dihydroxyvitamin D, 1,25D) locally from circulating calcifediol in response to pathogen threat and an immune-specific cytokine network. Calcitriol regulates gene expression through its binding to the vitamin D receptor (VDR), a ligand-regulated transcription factor. The hormone-bound VDR induces the transcription of genes integral to innate immunity including pattern recognition receptors, cytokines, and most importantly antimicrobial peptides (AMPs). Transcription of the human AMP genes β-defensin 2/defensin-β4 (*HBD2/DEFB4*) and cathelicidin antimicrobial peptide (*CAMP*) is stimulated by the VDR bound to promoter-proximal vitamin D response elements. HDB2/DEFB4 and the active form of CAMP, the peptide LL-37, which form amphipathic secondary structures, were initially characterized for their antibacterial actively. Notably, calcitriol signaling induces secretion of antibacterial activity in vitro and in vivo, and low circulating levels of calcifediol are associated with diverse indications characterized by impaired antibacterial immunity such as dental caries and urinary tract infections. However, recent work has also provided evidence that the same AMPs are components of 1,25D-induced antiviral responses, including those against the etiological agent of the COVID-19 pandemic, the SARS-CoV2 coronavirus. This review surveys the evidence for 1,25D-induced antimicrobial activity in vitro and in vivo in humans and presents our current understanding of the potential mechanisms by which CAMP and HBD2/DEFB4 contribute to antiviral immunity.

## 1. Overview of Vitamin D Signaling in Innate Immunity

Vitamin D was discovered as the cure for nutritional rickets, which arises from insufficient uptake of dietary calcium. We have known since the 19th century that cod liver oil has anti-rachitic activity. In a famous experiment performed 100 years ago, McCollum and coworkers showed that heated, oxidized cod-liver oil, in which vitamin A had been destroyed, lost the capacity to prevent xerophthalmia but could still cure rickets in rats [1,2]. Subsequent work revealed that seco-steroidal vitamin D3 could be produced in skin in the presence of sufficient ultraviolet B irradiation via the photochemical and thermal conversion of the conjugated double bonds of the cholesterol precursor, 7-dehydrocholesterol [3]. Whether it is obtained from dietary sources, supplementation or cutaneous photo-conversion, vitamin D3 must undergo sequential hydroxylation to become biologically active. The first occurs at the 25 position of the cholesterol side chain and is catalyzed largely but not exclusively by CYP2R1 [4,5]. Calcifediol (25-hydroxyvitamin D3, 25D) is the major circulating metabolite and the predominant indicator of vitamin D status. Calcifediol is modified by 1α-hydroxylation catalyzed exclusively by CYP27B1 to produce the active form, calcitriol (1,25-dihydroxyvitamin D3, 1,25D). Calcitriol binds to the nuclear vitamin D receptor (VDR), a ligand regulated transcription factor [6], and thus exerts its physiological actions by directly or indirectly regulating gene expression.

Unsurprisingly, given the origins of its discovery, vitamin D was studied throughout much of the 20th century as a factor critical for normal calcium homeostasis. Notably, expression of CYP27B1 in the kidney is tightly regulated by calcium regulatory inputs such as parathyroid hormone and FGF23 [6]. However, over the years, evidence accumulated that the physiological actions of vitamin D signaling were not limited to control of calcium status. Study of these “non-classical actions” of vitamin D was spurred on by the observations that the VDR and CYP27B1 were expressed in several tissues not implicated in calcium homeostasis [7]. Perhaps the best-established non-classical role of vitamin D3 is in the immune system. Both the VDR and CYP27B1 are expressed in cells of the innate and adaptive arms of the immune system, and, critically, expression of CYP27B1 in immune cells in humans is regulated by pathogen detection and a complex cytokine network [8,9,10,11], thus, independently of calcium homeostasis. The pathogen-dependent local production of calcitriol from calcifediol in immune cells represents one of the key pieces of evidence for a role of vitamin D in immune system regulation. 

For surveys of the role of vitamin D in regulation of adaptive immunity, readers are referred to the following reviews [12,13]. I will focus here on the innate arm of the immune system where work in the last few decades has shown that calcitriol signaling regulates multiple aspects of innate immunity [13]. These include the expression of so-called pattern recognition receptors (PRRs). PRRS are “first-responders” that recognize molecular motifs associated with pathogens, such as bacterial cell wall, cell membrane and tail components, as well as various forms of nucleic acid associated with invading viral genomes [14]. We have known for several years that calcitriol stimulates the transcription of the gene encoding CD14, a cofactor of the PRR toll-like receptor 4 (TLR4) [15], and more recent work has documented induction by calcitriol of the genes encoding PRRs TLR2 and NOD2 [16,17]. Vitamin D signaling also stimulates cytokine responses in innate immune cells exposed to pathogen, including interleukin 1β (IL1β) and IL8/CXCL8, a neutrophil chemokine [18], thus promoting communication between pathogen-exposed innate immune cells and other components of the immune system. Finally, and most interestingly, the calcitriol-bound VDR directly induces the transcription of genes encoding antimicrobial peptides (AMPs). Proximal promoter sequences of the human cathelicidin antimicrobial peptide (*CAMP*) and human beta-defensin 2 (*HBD2/DEFB4*) genes contain consensus vitamin D response elements (VDREs), binding sites for the VDR [19]. Collectively, these results reveal that calcitriol signaling induces the expression of numerous components of a robust innate immune response.

The goals of the sections below are to delineate the evidence accumulated from preclinical and clinical studies that calcifediol status regulates the production of antimicrobial peptides. Initial focus will be on the antibacterial activity of calcitriol-regulated AMPs, as the evidence for a relationship between calcifediol status and regulation of antibacterial activity in vitro and in vivo is most strongly established. I will then focus on the expanding data supporting roles for calcitriol-induced AMP expression in antiviral immunity. Wherever possible, primary sources obtained through numerous keyword searches in multiple databases are used as reference material, except for sentences that introduce broad subject areas, where comprehensive reviews on the subject are more appropriate. As the review takes a historical approach to vitamin D biology in general, and to the accumulating evidence for vitamin D-regulated AMP activity specifically, references range from the 1920s to the present day. Note that some references describing rapidly emerging evidence for antiviral activity of AMPs were taken from manuscripts deposited online but not yet peer-reviewed. These are described in the text as preprints or material yet to be subjected to peer review and their contents should be treated with caution.

## 2. Regulation of AMP Responses by Calcitriol Signaling In Humans In Vitro and In Vivo

Antimicrobial peptide is a kind of catch-all term that refers to AMPs that may have antibacterial, antiviral or antifungal activities [20]. Cathelicidins are well conserved throughout evolution [21] and were so-named because they have a conserved N-terminal domain that was first characterized for its efficacy in inhibition of cathepsin L. While many species have multiple cathelicidins, humans have only one. The cationic active human peptide LL-37, which is cleaved from the 18 KDa precursor form hCAP18, has no secondary structure in solution, but forms an amphipathic α-helix when it comes in contact with hydrophobic surfaces such as bacterial membranes [21]. HBD-2 is a 64 amino acid cationic peptide containing antiparallel β-sheets. Like cathelicidin, its expression is induced by wound healing in the skin and it is present in a number of epithelial tissues, notably the intestine [21,22]. Because of their amphipathic natures, both cathelicidins and defensin peptides have been best characterized for their bacterial membrane-disrupting capacities driven by interactions with hydrophobic and phospholipid components [21].

From a historical perspective, the discovery that calcitriol induced the transcription of genes encoding AMPs was compelling. It provided a molecular basis for links between sun exposure in the form of heliotherapy [23], as well as the use of cod liver oil, in treatment of tuberculosis, either as a respiratory infection, as lupus vulgaris (cutaneous TB) or scrofula (cervical TB lymphadenopathy caused by *Mycobacterium tuberculosis*) [24,25,26]. The induction of *CAMP* expression by calcitriol also provided links between vitamin D, cathelicidin and epithelial wound healing [27,28,29]. Whereas profound vitamin D deficiency causes rickets in humans and several animal models, many of the effects of vitamin D on innate immune signaling appear to be human/primate-specific. Gombart and coworkers showed that the VDRE in the human *CAMP* promoter is imbedded in a human/primate specific Alu repeat transposable element that appears to have been inserted before the old world-new world primate split [30,31]. Similarly, the VDRE in the *HBD2/DEFB4* promoter is not conserved in rodents, and no evidence for induction by calcitriol of mouse homologues of *CAMP* or *HBD2* was observed in mouse cells of epithelial or myeloid origin [32]. Unlike treatment of human cells [19,32], exposure of mouse cells to calcitriol did not induce release of antibacterial activity [32]. Importantly, experiments in human macrophages showed the engagement of the PRR TLR2 stimulation expression of CYP27B1, which converted calcifediol to calcitriol and induced transcription of the *CAMP* gene [9]. Further, the degree of *CAMP* induction in TLR2-stimulated macrophages cultured in the presence of human serum was dependent on calcifediol status; physiologically deficient serum was defective in stimulating CAMP transcription, underlining the importance of calcifediol status in vitamin D-regulated innate immune responses [9].

Of the two AMP genes initially identified as targets of calcitriol signaling in vitro, the regulation of *CAMP* has since attracted considerably more interest. Calcitriol induced *CAMP* transcription several-fold in epithelial and myeloid cells in vitro, whereas it was more efficacious at enhancing *HBD2/DEFB4* expression stimulated by other signals such as IL-1β [19], thus serving more as a secondary regulator of the gene. Since then, numerous studies have linked CAMP expression to calcifediol status and anti-bacterial immunity in humans. In an intervention trial, vitamin D supplementation (1000 IU/day for 90 days) enhanced antimicrobial activity in lung airway surface liquid (ASL) [33]. More intriguingly, ASL antimicrobial activity was found to be more robust in summer/fall than in winter-spring, consistent with seasonal fluctuations in circulating calcifediol levels. Moreover, an antibody against LL-37, the active form of cathelicidin, blocked antimicrobial activity in ASL, and the seasonality of antimicrobial activity was eliminated by vitamin D supplementation [33].

The above findings are reinforced by links between calcifediol status and indications associated with defects in anti-bacterial innate immunity. One such condition is dental caries, which is caused by oral bacterial flora. Several clinical studies, including an umbrella study of systematic reviews, have linked low calcifediol levels to increased risk of caries [34,35,36], and a systematic review of randomized controlled trials estimated that vitamin D supplementation reduced rates of dental caries by about 50% [37]. At a more molecular level, a positive correlation was observed between CAMP levels in saliva and calcifediol status [35], and subsequent work showed that CAMP/LL-37 is efficacious against bacterial species such as *Streptococcus mutans*, a major constituent of plaque [38].

Poor calcifediol status is also a risk factor for urinary tract infections (UTI). Vitamin D signaling induces *CAMP* expression in the urinary bladder [39], and urinary LL-37 but not HBD2/DEFB4 levels correlate with calcifediol status in children [40]. Several clinical studies have linked vitamin D deficiency with increased rates of UTI in all age groups but particularly in children [40,41,42,43]. A recent meta-analysis covering 9 studies and 1921 total participants, including 580 with UTI, revealed lower calcifediol levels in the UTI group [44]. Moreover, there was a significant association of vitamin D insufficiency with increased risk of UTI (pooled OR = 3.01, 95%CI = 2.31–3.91). The same study also confirmed the strong association between UTI and vitamin D deficiency in children (OR = 4.78, 95%CI = 3.08–7.44, *p* < 0.001) [44]. UTI are associated to other conditions, such as non-alcoholic fatty liver disease (NAFLD), which is also correlated with calcifediol deficiency [45,46]. While causal links between low calcifediol levels and NAFLD should be treated with caution because both are associated with obesity and sedentary lifestyles, there is emerging evidence that vitamin D deficiency may exacerbate NAFLD. One area of intense interest in the field is the association between altered gut microbiota and their metabolites and NAFLD [47]. Indeed, vitamin D status may influence the severity of NAFLD through its effects on intestinal innate immunity and gut microbial composition [48]. There is also some indirect support for a potential role of vitamin D-regulated AMPs in attenuating NAFLD. In high-fat diet-treated diabetic mice, lentiviral overexpression of cathelicidin reduced hepatic steatosis and increased lean mass in mice [49].

Another indication linked to low calcifediol levels and impaired intestinal innate immunity is Crohn’s disease (CD), a relapsing, recurring inflammatory bowel condition [50]. Several small clinical studies exploring the relationship between CD disease activity and vitamin D supplementation or status have been published. These have recently been reviewed in two meta-analyses, both of which found a decrease in relapse rates in vitamin D-supplemented CD patients relative to controls [51,52]. LL-37 is of particular interest in CD because of its role in epithelial wound healing and maintenance of intestinal barrier integrity. One small intervention trial showed that vitamin D supplementation (2000 IU/day) increased LL-37 levels and maintained intestinal integrity [53], which is consistent between the inverse correlation observed between LL-37 levels and disease activity in CD patients [54]. It is also noteworthy that genome-wide association studies have revealed that the gene encoding the PRR NOD2 is a CD susceptibility locus [55,56], as is the *HBD2/DEFB4* gene [57]. The *HBD2/DEFB4* has undergone a series of gene-duplication events, and low copy number correlated with increased risk of colonic CD [57]. This is of interest because *NOD2* and *HBD2/DEFB4* sit at the extremities of an innate immune signaling pathway, and expression of both genes is induced by calcitriol [17]. One study found that, in CD patients with circulating bacterial DNA, levels of HBD-2 and LL-37 were dependent on patients having a wild-type NOD2 status [58]. Taken together, the above findings provide compelling evidence that calcifediol levels correlate with expression and function of AMPs in vivo in humans.

## 3. Calcitriol Signaling, AMPs and Antiviral Immunity

Clinically, there is evidence for immune protection induced by calcitriol against several viral pathogens, including HIV, hepatitis viruses, and several respiratory viruses [59]. A meta-analysis of clinical data published in 2017 provided compelling evidence that daily or weekly vitamin D supplementation reduced the risk of acute respiratory tract infections, particularly in individuals with the poorest vitamin D status [60]. An expanded follow-up to this study published in 2021 was less emphatic. It found substantial heterogeneity in results across trials but still concluded that vitamin D supplementation conferred a small risk reduction, but only if taken daily [61]. While these types of studies are important, interpretation of vitamin D supplementation trials should be treated with caution because vitamin D is a nutrient and not a drug and is available from the diet and from sun exposure, complicating placebo wings. As a result, many intervention trials have been conducted on vitamin D sufficient populations. It is therefore important to examine all of the data, including that arising from preclinical studies, which have provided evidence for multiple mechanisms by which calcitriol signaling could enhance antiviral immunity.

There are remarkable parallels between the mechanisms of antiviral and anti-bacterial innate immune responses. As with anti-bacterial innate responses, invading viral pathogens are first detected through specific PRRs. Engagement of PRRs stimulates signaling cascades that culminate in induction of cytokines, type 1 interferons and antiviral effectors [62,63]. Invading viral genomes are detected via intracellular or endosomal PRRs such as TLR3, TLR7 or TLR9, which detect various forms of nucleic acid [62]. Downstream signaling pathways are funneled through a limited number of transcription factors, including interferon regulatory factor, AP-1, and NF-κB family members [64], which induce interferon signaling and subsequent generation of antiviral effectors. As developed below, calcitriol-induced AMPs are implicated in many aspects of antiviral response, including enhancement of TLR signaling. Many respiratory viruses target lung epithelial cells, which are highly vitamin D-responsive. For example, exposure of primary human lung epithelial cells to dsRNA, which mimics viral genomes, enhanced the expression of CYP27B. In dsRNA-stimulated epithelial cells, robust *CAMP* expression was induced in the presence of calcifediol [65]. Similarly, respiratory syncytial virus (RSV) infection of bronchial epithelial cells induced 1α-hydroxylase activity [66]. Thus, calcifediol is converted to calcitriol locally in response to infection in lung epithelial cells.

**Figure 1:**
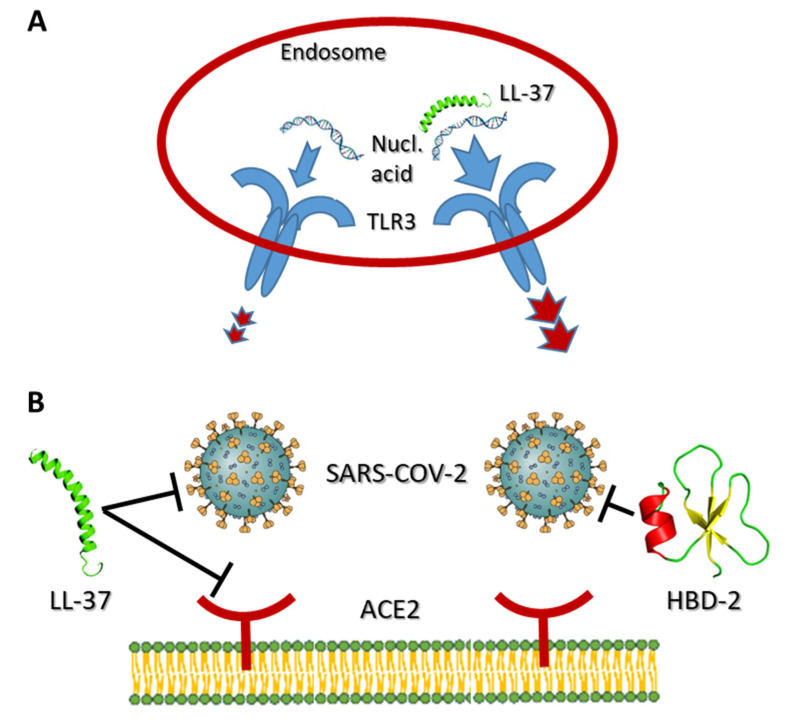
Contributions of vitamin D-induced AMPs to antiviral immunity. (**A**) Interaction of the positively charged LL-37 with nucleic acid augments binding and signaling through nucleic acid-sensing TLRs. TLR3 is used as an example. (**B**) Interactions of LL-37 and HBD-2 with SARS-COV-2 spike protein and/or cell surface receptor ACE2 block viral entry.

Induction of *CAMP* gene transcription during infection has emerged as a multifaceted component of antiviral responses regulated by vitamin D. Evidence is accumulating from in vitro studies with a number of respiratory viruses that AMPs intervene at different stages throughout viral life cycles. Importantly, LL-37, as a positively charged peptide, binds to negatively charged nucleic acids, protecting them from extracellular nuclease degradation. This enhances endocytosis and detection by a number of endosomal, nucleic acid-sensing TLRs (Figure 1A). Depending on the cell type and the nucleic acid species, LL-37 can augment signaling through TLR3, TLR7 or TLR9 leading to elevated interferon- responses [67,68,69,70]. There has been some suggestion that the DNA-binding activity of LL-37 is an off-shoot of its role in wound healing, as it may play a role in expedited clearance of nucleic acid released from dying cells [70]. There is also evidence that secreted LL-37 may selectively regulate nucleic acid-sensing TLR expression. For example, exposure of normal keratinocytes to LL-37 strongly induced expression of TLR9 by an unknown mechanism, while TLR3 expression was unaffected [71].

Another mechanism through which LL-37 and other AMPs may intervene to block viral infections is through direct interaction with viral particles, destabilizing their envelopes [72]. Influenza A (InA) virus is a highly contagious pathogen responsible for annual global outbreaks [73]. In vitro studies have suggested that LL-37 interacts directly with InA capsids, and electron microscopy data has provided evidence that this disrupts the integrity of viral membranes [74]. Another target of LL-37 is RSV, which is one of the most common pathogens infecting children and adults. Infection leads to upper and lower respiratory tract illness, which in severe cases progresses to pneumonia, respiratory failure and death [75]. Calcitriol-stimulated CAMP expression attenuated RSV-induced apoptosis and replication in vitro and suppressed the assembly of viral particles [76]. Further work by the same group showed that LL-37 interacts directly with RSV, damaging the viral envelope. More importantly, in the same study, exogenous LL-37 was protective in a murine model of RSV infection in vivo, and elevated LL-37 levels in nasal samples were associated with protection in a human model of RSV infection [77]. Results with rhinovirus (RV) are mixed. While one study found no effect of calcitriol on RV replication in bronchial epithelial cells [78], another, using primary cystic fibrosis bronchial cells, found that calcitriol suppressed RV load via induction of CAMP [79]. In addition, treatment with calcitriol of RV-infected cells inhibited viral replication and release [66].

## 4. LL-37, HBD-2 and SARS-CoV2

With the arrival of the COVID-19 pandemic, links between vitamin D status and outcomes of SARS-CoV2 coronavirus infections have attracted intense interest [80]. Recent studies, one of which is a preprint [81], report in silico molecular docking approaches with the SARS-CoV2 receptor binding domain (RBD) and predict that LL-37 and HBD-2 interact strongly with the RBD [81,82], and biophysical assays using both peptides supported these findings [82,83] (Figure 1B). Two papers studied the antiviral properties of LL-37 and HBD-2 using SARS-CoV2 pseudo-virion approaches. One, examining LL-37, provided evidence that it can bind to the spike protein and cloak the cellular SARS-CoV2 receptor ACE2 [84] (Figure 1B). Consistent with these findings, LL-37 was an efficacious inhibitor in mouse models of infection by spike protein-expressing pseudo-virions [83]. Similarly, HBD-2 selectively blocked infections by spike protein-expressing pseudo-virions, but not those expressing vesicular stomatitis virus glycoproteins [83]. This latter finding of selectively is encouraging as it suggests that AMPs are not generally “sticky” in these types of assays. However, at the time of writing, the evidence for anti-SARS-CoV2 activity of LL-37 is stronger than that for HBD-2, as neither HBD-2 study [81,83] has been published in peer-reviewed form. Strikingly, in other findings yet to be peer-reviewed, use of an oral, recombinant form of LL-37 in a small-scale, single-arm exploratory clinical trial in patients infected with SARS-CoV2 in China did not report any adverse reactions but did note some relief of gastrointestinal symptoms [85]. This study is intriguing, but should be treated with caution—it will be of interest to see it in its peer-reviewed form.

## 5. Conclusions

Since the discovery of calcitriol-regulated AMP gene transcription in vitro in 2004, numerous findings have accumulated supporting the notion that calcifediol status affects antimicrobial defenses in vivo in humans. The bulk of the studies on the antimicrobial functions of LL-37 and HBD-2 have focused on their antibacterial activities, and the evidence in vivo in humans for their roles, particularly that of LL-37, in antibacterial defenses is compelling. Most of the data suggesting that LL-37 and HBD-2 are components of calcitriol-induced antiviral defenses is derived from in vitro studies, but is supported by some experiments in animal models. Given the recent advent of the COVID-19 pandemic, the evidence for roles of LL-37 and HBD-2 in antiviral responses to SAR-CoV-2 infection is best described as emerging, but definitely merits further investigation.

## Data Availability

Not applicable.
